# Psychological, Symptom-Related, and Lifestyle Predictors of Health-Related Quality of Life in Hungarian Women with Endometriosis

**DOI:** 10.3390/jcm14197004

**Published:** 2025-10-03

**Authors:** Zsófia Kovács-Szabó, Pongrác Ács, Viktória Prémusz, Alexandra Makai, Márta Hock

**Affiliations:** 1 Doctoral School of Health Sciences, Faculty of Health Sciences, University of Pécs, 7622 Pécs, Hungary; viktoria.premusz@etk.pte.hu; 2 Institute of Physiotherapy and Sport Science, Faculty of Health Sciences, University of Pécs, 7622 Pécs, Hungary; pongrac.acs@etk.pte.hu (P.Á.); alexandra.makai@etk.pte.hu (A.M.); hock.marta@etk.pte.hu (M.H.); 3 Physical Activity Research Group, Szentágothai Research Centre, University of Pécs, 7622 Pécs, Hungary

**Keywords:** endometriosis, pelvic pain, quality of life, mental health, physical activity, pain-related self-efficacy

## Abstract

**Background**: This study was a cross-sectional online survey aimed at examining health-related quality of life and the effect of different symptoms and lifestyle factors on health-related quality of life in a sample of women with endometriosis in Hungary. **Methodology**: A cross-sectional online survey was carried out in a sample of women with endometriosis. Self-edited and Hungarian versions of validated questionnaires were used to assess health-related quality of life (Sf-36-Health Survey—SF-36), pain (Numeric Rating Scale-NRS), effect of pelvic pain on everyday life (Pelvic Pain Impact Questionnaire—PPIQ), perceived stress levels (Perceived Stress Scale—PSS), and physical activity (Global Physical Activity Questionnaire—GPAQ). Data analysis was conducted using IBM SPSS Statistics 28.0, and the level of significance was set at *p* < 0.05. Multivariate linear regression analysis was performed to examine the effect of different lifestyle factors, pain-related, and physical symptoms on the participants’ health-related quality of life (HrQoL). **Results**: The health-related quality of life of Hungarian women with endometriosis in our sample was significantly lower than the latest Hungarian normative values. Multiple linear regression analyses revealed that psychological, pain-related, and lifestyle factors significantly predicted HrQoL across SF-36 subscales in women with endometriosis (all models, *p* < 0.001; Adjusted R^2^ = 0.274–0.654). Pain self-efficacy (PSEQ) was a consistent positive predictor that was significantly associated with better scores in five SF-36 domains, including physical and social functioning. Perceived stress (PSS) is a strong negative predictor that particularly affects emotional well-being, energy/fatigue, and social functioning. Pain interference (PPIQ) was linked to poorer HrQoL in seven out of eight SF-36 domains, while average pain intensity (NRS) negatively predicted Physical Functioning and General Health. Vigorous physical activity was positively associated with Social Functioning, whereas moderate activity had no significant effect. Among the demographic factors, only age was negatively associated with Physical Functioning; BMI and education were not significant predictors. **Conclusions**: Psychological, lifestyle, and symptom-related factors play key roles in health-related quality of life among women with endometriosis. Self-efficacy was a strong positive predictor, whereas perceived stress and pain interference were linked to poorer outcomes. High-intensity physical activity supported better social functioning. These findings highlight the need for multidisciplinary interventions targeting psychological support, pain management, and physical activity to improve quality of life in this population.

## 1. Introduction

Endometriosis is a chronic, progressive gynaecological disorder characterized by the presence of endometrial-like tissue outside the uterine cavity, and it affects approximately 6–13% [1] (114–247 million) women worldwide [2,3,4].

In Hungary, outpatient data indicate age-specific prevalence rates of 275.27 per 100,000 among women aged 20–29, 694.96 per 100,000 among those aged 30–39, and 438.94 per 100,000 among the 40–49 age group. A recent study reported that €1.91 million was spent on the treatment of endometriosis in Hungary in 2019, indicating the significance of the disease [5]. The impact on work productivity is substantial, with an estimated average loss of 11 working hours per week among women with endometriosis [6,7].

Endometriosis is recognized as both a predisposing and perpetuating factor for chronic pelvic pain, which significantly affects all aspects of life and further reduces health-related quality of life [8]. The main physical symptoms of endometriosis are pelvic pain and other pain conditions such as dyspareunia, dyschezia, and dysmenorrhea; however, the disease is often associated with many other physical symptoms such as bloating, heavy period, weakness, fatigue, low back pain, and even frequent headaches [9].

Beyond the debilitating physical symptoms and the significant financial and work-related burden, endometriosis significantly impairs health-related quality of life and negatively influences mental health [10]. The psychological consequences—such as anxiety, depression, irritability, elevated stress levels, and even severe fatigue—could be as debilitating as the physical symptoms themselves [11,12,13].

Endometriosis leads to limitations not only in quality of life, as noted in the recent ESHRE guideline [14], but in daily functioning, including family responsibilities and social relationships; for example, absence from family or social events could also be closely connected to endometriosis [15,16,17].

In recent years, there has been growing recognition among Hungarian scientists that addressing the impact of gynaecological conditions [18], including endometriosis, on an individual’s health-related quality of life is essential. Psychosocial factors such as self-efficacy, perceived stress, and pain catastrophizing also play a crucial role in the everyday management of the disease [19].

### Objective

This study aimed to examine the effects of various physical, lifestyle, and psychological factors on health-related quality of life in women living with endometriosis in a Hungarian population.

## 2. Materials and Methods

### 2.1. Study Design and Sample

The present study employed a cross-sectional design using an open online survey disseminated via social media platforms within endometriosis support groups in Hungary between January and June 2023. Female participants aged 18–45 years with a self-reported medical diagnosis of endometriosis were recruited through a convenience sampling method. Before participation, participants received information regarding the study objectives and estimated duration. Exclusion criteria included the absence of an endometriosis diagnosis, current pregnancy, presence of severe medical conditions (e.g., malignant tumors or untreated psychiatric disorders), age below 18 or above 50 years, postmenopausal status, recent surgical intervention within the past six months, and lack of informed consent.

A post hoc power analysis was performed using G*Power 3.1.9.7 for Windows [20] to evaluate the adequacy of the sample size for the multiple linear regression model, which included nine predictors and a total sample of 262 participants. The analysis yielded a noncentrality parameter (λ) of 39.30, a critical F-value of 1.92, numerator degrees of freedom = 9, and denominator degrees of freedom = 252. The calculated statistical power was 0.997, indicating a very high probability of detecting a true effect at the conventional alpha level of 0.05. These results suggest that the sample size is sufficient to support the validity of the regression findings.

In our study, we adhered to both the Strengthening the Reporting of Observational Studies in Epidemiology (STROBE) [21] and CHERRIES (Checklist for Reporting Results of Internet E-Surveys) [22] guidelines to ensure comprehensive and transparent reporting of our observational and web-based survey methodologies.

### 2.2. Instruments

We used a self-edited questionnaire to collect data on anthropometric (weight and height) and sociodemographic characteristics (age, residence, marital status, and educational level), and information regarding obstetric and gynecological history (endometriosis diagnosis, symptoms, and other gynecological symptoms such as inflammation) and lifestyle factors (diet and alternative methods used for relieving endometriosis symptoms). Additionally, we administered the validated Hungarian versions of several questionnaires during both evaluations: the Pelvic Pain Impact Questionnaire (PPIQ), the Pain Self-Efficacy Questionnaire (PSEQ), the Perceived Stress Scale (PSS-10),) the Numeric Rating Scale (NRS), the Global Physical Activity Questionnaire (GPAQ), and the 36-item Short Form Health Survey (SF-36). The first version of the questionnaire was tested on volunteers before distribution.

#### 2.2.1. Pelvic Pain Impact Questionnaire

The self-administered PPIQ [23] is a simple questionnaire that aims to assess how pelvic pain affects daily life. The questionnaire consists of 8 mandatory and 2 optional questions, each rated on a 5-point Likert scale. The total score ranges from 0 to 32, with higher scores indicating a more significant impact of pelvic pain on the individual’s daily activities.

#### 2.2.2. Pain Self-Efficacy Questionnaire

The self-administered PSEQ [24,25] is a specific questionnaire developed to assess pain-related self-efficacy at present in people with chronic pain, aimed at assessing how confident the participants feel in performing everyday activities despite experiencing pain. The questionnaire includes 10 questions rated on a 7-point Likert scale. The score is calculated by summing the responses; the maximum score is 60, showing excellent pain-related self-efficacy.

#### 2.2.3. Perceived Stress Scale-14

The PSS-14 [26,27] is a self-administered questionnaire developed to assess stress levels and “the degree to which individuals appraise situations in their lives as stressful”. The scale includes fourteen questions, consisting of five answer options related to frequency. Higher scores indicate higher stress levels, with 56 indicating the highest possible score.

#### 2.2.4. 36 Item Short-Form Health Survey (SF-36)

The 36-Item Short-Form Health Survey [28,29,30] is designed to assess participants’ health-related quality of life across eight health concepts. This measurement tool is suitable for evaluating the health-related quality of life of individuals with endometriosis [31]. Its scoring process consists of two steps. First, scores are summed for each domain, resulting in scores that range from 0 to 100, where 0 indicates the lowest health-related quality of life and 100 represents the highest. Then, the items within each scale are averaged to create eight domains: Physical functioning (10 items), Bodily pain (2 items), Role limitations due to physical health problems (4 items), Role limitations due to emotional problems (3 items), Emotional well-being (5 items), Social functioning (2 items), Energy/fatigue (4 items), and General health perceptions (6 items).

#### 2.2.5. Numeric Pain Rating Scale (NRS)

The scale [29,32,33] utilized in this study is a well-recognized and validated instrument in the literature, specifically designed to assess subjective pain levels on a scale from 0 to 10. On this scale, 0 indicates no pain, while 10 signifies the most severe pain imaginable. Participants were asked to rate their average and maximum pain levels over the past four weeks, as well as their current pain intensity.

#### 2.2.6. General Physical Activity Questionnaire (GPAQ)

The Questionnaire [34,35] developed by the World Health Organization (WHO) is a standardized instrument designed to assess physical activity patterns in adult populations. It collects data across three domains: work-related physical activity, travel to and from places, and recreational activities, including moderate and vigorous sport. The GPAQ collects information on the frequency (days per week) and duration (minutes per day) of moderate- and vigorous-intensity physical activity, as well as sedentary behavior. The tool enables the estimation of total physical activity in minutes per week.

#### 2.2.7. Ethical Considerations

The study was conducted anonymously, with all participants required to give informed consent after receiving detailed information about the research. They could only proceed with the questionnaire after providing their consent. Participation remained anonymous, as the study was conducted through an online questionnaire that did not require any registration or identification. Data was collected using a research ID to ensure participant privacy. Ethical approval for the study was obtained from the Institutional Review Board of the Faculty of Medicine, University of Pécs, in 2023 (no.: 9534-PTE 2023; Clinical trial number: NCT05863663), and all methods used in this study were performed in accordance with the relevant guidelines and regulations.

#### 2.2.8. Statistical Analysis

Data analysis was conducted using IBM SPSS Statistics 28.0 (IBM Corp., Armonk, NY, USA). Descriptive statistics included mean, standard deviation, median, and interquartile range. The distribution of the variables was examined by using the Kolmogorov–Smirnov test. Multivariate linear regression analysis was conducted to examine the associations between the independent variables and the outcome measures. All predictors were entered simultaneously using the Enter method. For each model, we report the standardized regression coefficients (β) with their 95% confidence intervals, the R^2^ and adjusted R^2^ values, and the F- and *p*-values for overall model fit. One-sample *t*-tests were conducted to assess the differences between the Hungarian normative values and the current sample’s HRQoL scores, and Cohen’s d was calculated to estimate the effect size with 0.2, 0.5, and 0.8 interpreted as small, medium, and large effects.

## 3. Results

### 3.1. Descriptive Data

The questionnaire was distributed to approximately 1500–2000 women experiencing endometriosis through various Hungarian online groups focused on expert guidance and self-help. Of all the individuals contacted, 277 completed the questionnaire; however, 15 were subsequently excluded from the study. The reasons for exclusion included three cases of ongoing pregnancy, eight respondents who did not have endometriosis, and four participants who were older than 50 years. Consequently, 262 women with endometriosis completed the questionnaire and participated in the study. Of the sample, 76.3% underwent at least one endometriosis surgery, and 23.7% had endometriosis diagnosed through MR and US examinations by specialist physicians. Moreover, 90.5% of the participants had pelvic pain at least 1 or 2 times/month, and the average duration of pelvic pain was 9.2 ± 7.7 years. The average age of participants was 34.39 years, with a standard deviation of 6.68 years. The sociodemographic, obstetric, and gynaecological characteristics of the participants are presented in Table 1.

The average scores of the applied measurement tools are presented in Table 2. Notably, 188 (72%) participants reported high levels of perceived stress.

### 3.2. Comparison of Current SF-36 Domain Scores with Hungarian Normative Values

Initially, we compared the health-related quality of life of the participants with Hungarian normative values. To achieve this, we used the average scores of the SF-36 for Hungarian women aged 18 to 44 and then compared these mean scores with the subscale scores from our sample. The results are presented in Table 3 below. The average scores in our sample are significantly lower than the Hungarian normative values (*p* < 0.001).

### 3.3. Impact of Physical and Mental Health on Health-Related Quality of Life

To assess the relationships between psychological, pain-related, and lifestyle factors and health-related quality of life in women with endometriosis, multiple linear regression analyses were conducted using the eight subscales of the SF-36 as dependent variables.

Each model included the following predictors: psychological variables: Pain Self-Efficacy Questionnaire (PSEQ), Perceived Stress Scale (PSS14), physical symptom variables such as average pain intensity on the NRS, Pelvic Pain Impact Questionnaire (PPIQ), and controlling factors: age, BMI, educational level, and levels of physical activity (GPAQ moderate and intensive physical activity). All models were statistically significant (*p* < 0.001) and showed acceptable explanatory power, with Adjusted R^2^ values ranging from 0.274 (role limitations due to emotional problems) to 0.654 (body pain).

Table 4 shows the results of the regression analysis.

#### 3.3.1. Psychological Factors

As previously discussed, psychological constructs, particularly self-efficacy, are significant predictors of health-related quality of life (HrQoL). In the current analysis, self-efficacy, measured using the Hungarian version of the Pain Self-Efficacy Questionnaire (PSEQ-HU), demonstrated a consistent and positive association with multiple domains of the SF-36 health survey. Specifically, higher self-efficacy scores were significantly associated with better Physical Functioning (*p* = 0.015), Role Limitations due to Physical Health (*p* < 0.001), energy/fatigue (*p* = 0.004), Bodily Pain (*p* < 0.001), and Social Functioning (*p* < 0.001).

In addition, perceived stress, assessed using the Perceived Stress Scale (PSS14), was identified as a significant negative predictor across several SF-36 domains. Elevated stress levels were associated with poorer emotional well-being (*p* < 0.001), Role Limitations due to Emotional Health (*p* < 0.001), energy/fatigue (*p* < 0.001), General Health (*p* < 0.001), and Social Functioning (*p* < 0.001). These results suggest that the psychological burden of stress not only impacts mental health but also contributes to perceived limitations in energy and social roles.

#### 3.3.2. Impact of Pelvic Pain

The perceived impact of pelvic pain measured with the pelvic pain impact questionnaire (PPIQ) on daily activities emerged as a robust predictor of HrQoL, showing significant associations with seven out of eight SF-36 domains. Higher pain interference scores were associated with reduced functioning in the following domains: Physical Functioning (*p* = 0.015), Role Limitations owing to Physical Health (*p* < 0.001), emotional well-being (*p* = 0.019), role limitations owing to Emotional Health (*p* = 0.032), energy/fatigue (*p* = 0.006), Bodily Pain (*p* < 0.001), and Social Functioning (*p* < 0.001).

Furthermore, the average pain intensity, measured on a Numerical Rating Scale (NRS), was a significant negative predictor in two SF-36 domains: Physical Functioning (*p* = 0.028) and General Health (*p* = 0.014).

#### 3.3.3. Physical Activity

Among lifestyle-related factors, physical activity, particularly high-intensity exercise, had a significant positive association with Social Functioning (*p* = 0.034), suggesting the potential psychosocial benefits of regular intensive physical engagement. However, moderate-intensity physical activity did not yield significant associations with any of the SF-36 domains, indicating that intensity may be a key moderating factor in the relationship between physical activity and the perceived quality of life.

#### 3.3.4. Demographic Variables

Demographic characteristics, including age, body mass index (BMI), and educational attainment, were largely non-significant predictors of HrQoL in this sample. However, age demonstrated a modest yet statistically significant negative association with Physical Functioning (*p* = 0.031), suggesting a decline in perceived physical capability with increasing age. Neither BMI nor education level showed a statistically significant relationship with any of the SF-36 domains.

## 4. Discussion

Endometriosis and endometriosis-related pelvic pain significantly affect health-related quality of life (HRQoL) in Hungarian women. This was confirmed in our current study, as quality of life indicators in our sample differed negatively and significantly from Hungarian normative values.

To identify potential contributors to reduced HRQoL, this study examined the associations between psychological, symptom-related, lifestyle, and sociodemographic factors and HRQoL in a Hungarian sample of women diagnosed with endometriosis. Our findings demonstrate that psychological factors, particularly self-efficacy and perceived stress, play a prominent role in predicting multiple dimensions of HRQoL as measured by the SF-36. Additionally, the burden of chronic pelvic pain in this population has emerged as a significant predictor in several domains. In contrast, the demographic variables showed limited or no predictive value.

These findings highlight the importance of self-efficacy beliefs in the subjective evaluation of functioning, vitality, and social participation of individuals with chronic symptoms. Consistent with prior research, self-efficacy was found to be a strong positive predictor of HRQoL [36,37]. In our study, higher levels of pain self-efficacy were associated with improved physical functioning, greater energy consumption, reduced role limitations due to physical health, and better social participation. These findings reinforce the theory of self-efficacy [38], which shows that individuals’ beliefs in their ability to manage symptoms and daily tasks significantly influence their psychological and physical well-being. In chronic conditions such as persistent pelvic pain in endometriosis, enhancing self-efficacy may serve as a protective factor against the deterioration of quality of life in this patient group.

Conversely, perceived stress was negatively associated with HRQoL across a broad spectrum of SF-36 domains, particularly emotional well-being, general health, and fatigue. This aligns with the existing literature on other chronic diseases, demonstrating that higher stress levels exacerbate both mental and physical HRQoL outcomes and often mediate the relationship between illness burden and psychosocial functioning. Interestingly, perceived stress also significantly predicted social functioning, suggesting that stress may influence interpersonal relationships and engagement, possibly through withdrawal, emotional exhaustion, or reduced social support. Unfortunately, patients with endometriosis often show elevated perceived stress levels [36,39].

Regarding physical symptom burden, both the impact of pelvic pain on daily life and average pain intensity emerged as significant negative predictors of HRQoL, similar to the findings from previous endometriosis-related studies [40,41]. In our study, the impact of pelvic pain on everyday life, rather than pain intensity alone, accounted for greater variance across emotional, physical, and social domains. This indicates that a higher reported pain intensity correlates with greater functional limitations and a more negative overall health perception.

These results support the notion that the perceived impact of pain in daily activities may be more consequential to quality of life than pain intensity per se.

Notably, in the current study, physical activity—particularly at a vigorous intensity—was positively associated with social functioning. While this finding is in line with literature highlighting the psychological and social benefits of exercise [42,43], it is noteworthy that only vigorous physical activity, and not moderate activity, demonstrated a significant relationship in the current study. This phenomenon may be explained by the tendency of participants completing the GPAQ to overestimate the intensity of their physical activity, often classifying activities as vigorous even when they would be more accurately categorized as moderate [35,43].

Demographic variables such as age, BMI, and educational level did not appear to be significant predictors of HRQoL. Only age was found to be a modest predictor of physical functioning, suggesting that younger individuals may report better physical capabilities, independent of other psychosocial or behavioural factors. These results suggest that psychosocial and symptom-related variables may outweigh sociodemographic characteristics in predicting subjective quality of life outcomes in this population.

## 5. Study Strengths and Limitations

One of the main advantages of this study is its comprehensive approach to examining HRQoL, which includes psychological, behavioral, and physical symptom factors. The application of validated tools (SF-36, PSEQ-HU, and PSS) strengthened the reliability and applicability of the results. However, this study had several limitations. First, the cross-sectional nature of the study limits its ability to draw causal conclusions; longitudinal studies are necessary. Second, self-reported data, especially self-reported medical data, may be prone to recall and response bias, and observed effects and generalizability of the findings could be limited. Third, although the sample size was sufficient and statistical power was strong, the findings may only apply to similar clinical or demographic groups. Fourth, the GPAQ for measuring physical activity may have limitations due to the self-reported.

## 6. Future Directions

Future research should investigate the mediating and moderating roles of psychological constructs such as self-efficacy and perceived stress using longitudinal designs. Interventional studies are warranted to test whether improving self-efficacy or reducing stress leads to measurable improvements in the HrQoL. Additionally, further investigation into the role of physical activity intensity and social engagement in HrQoL maintenance may guide behavioral health interventions.

## 7. Conclusions

This study highlights the significant role of psychological and symptom-related factors in determining health-related quality of life among individuals with endometriosis in a Hungarian population. Self-efficacy emerged as a robust positive predictor, while perceived stress and pain-related interference were associated with a decreased quality of life across multiple physical, emotional, and social domains. Notably, high-intensity physical activity positively contributed to social functioning, suggesting the potential benefits of structured physical engagement.

Demographic variables, such as age, BMI, and educational level, were found to have limited predictive value, reinforcing the importance of targeting modifiable psychological and behavioral factors in clinical and public health interventions. These findings underscore the need for holistic, multidisciplinary approaches that incorporate psychological support, pain management, and physical activity promotion to enhance quality of life in this population.

## Figures and Tables

**Table 1:** Sociodemographic, obstetric, and gynaecological characteristics of the sample.

Variable	Final Sample n = 262	(%)
1. Marital status		
living alone	34	12.9%
living with partner	94	35.9%
married	129	49.3%
divorced	5	1.9%
2. Educational level		
elementary school	4	1.7%
secondary school	106	40.5%
higher education	151	57.8%
3. Work category		
sedentary	156	59.5%
light physical	58	22.1%
heavy physical	7	2.7%
standing	27	10.3%
unemployed	14	5.4%
4. Residence		
city	112	42.9%
village	43	16.5%
county seat	41	15.7%
capital	65	24.9%
5. Pelvic pain frequency		
every day	77	29.3%
3–4 times/week	42	16.0%
1–2 times/week	42	16.0%
3–4 times/month	25	9.6%
1–2 times/month	51	19.5%
no pain	25	9.6%
7. Had endometriosis surgery	184	70.2%

**Table 2:** Descriptive statistics of the measurement tools (SD = Standard Deviation, IQR = Interquartile Range).

Outcomes		Mean (SD)	Median	IQR Lower and Upper Bound	
Pain Self-Efficacy Questionnaire PSEQ—HU		36.18 ± 17.04	38.00	23.00	50.00
Pelvic Pain Impact Questionnaire—PPIQ		19.25 ± 10.82	17.00	9.00	23.00
Numeric Rating Scale—NRS, Current pain Intensity		4.09 ± 3.02	4.00	1.00	7.00
Numeric Rating Scale—NRS, Average pain intensity in the last month		5.09 ± 2.62	5.00	3.00	7.00
Numeric Rating Scale—NRS, Strongest pain intensity in the last month		6.79 ± 2.89	8.00	5.00	9.00
Perceived Stress Scale—PSS14		30.92 ± 8.72	31.00	26.00	36.00
Short-Form Health Survey—SF-36	Physical functioning	73.38 ± 23.59	55.00	95.00	80.00
	Role limitations due to physical health	43.87 ± 41.61	0.00	100.00	25.00
	Role limitations due to emotional problems	50.83 ± 44.39	0.00	100.00	66.66
	Energy/fatigue	45.06 ± 14.03	35.00	55.00	45.00
	Emotional wellbeing	46.16 ± 14.45	36.00	56.00	44.00
	Social functioning	62.26 ± 32.35	50.00	100.00	75.00
	Pain	46.33 ± 28.11	22.50	67.50	45.00
	General health	44.93 ± 23.74	25.00	60.00	40.00
GPAQ—Global Physical Activity Questionnaire	Moderate physical activity (min/week)	82.82 ± 128.46	0.00	120.00	40.00
	Vigorous physical activity (min/week)	59.08 ± 132.01	0.00	60.00	0.00

**Table 3:** A comparison of health-related quality of life values between our participants and the Hungarian normative values from 2025.

	Physical Functioning	Role limitations Due to Physical Health	Bodily Pain	General Health	Energy/Fatigue	Social Functioning	Role Limitations Due to Emotional Problems	Emotional Wellbeing
Hungarian normative values [30]	88.30	80.54	77.91	63.73	57.95	78.20	77.66	64.92
Current values	73.38	43.87	46.33	44.93	45.06	62.26	50.83	46.16
Level of significance (*p*)	*p* < 0.001	*p* < 0.001	*p* < 0.001	*p* < 0.001	*p* < 0.001	*p* < 0.001	*p* < 0.001	*p* < 0.001
t	−10.424	−14.215	−18.203	−13.587	−15.212	−7.999	−9.799	−21.277
Cohen’s d	−0.64	−0.87	−1.12	−0.95	−0.93	−0.49	−0.60	−1.30

**Table 4:** Summary of multiple linear regression analyses predicting SF-36 Subscale Score.

	SF36 Domains															
Predictors	SF36 PF		SF3 RP		SF36 EW		SF36 RE		SF36 VT		SF36 BP		SF36 GH		SF36 SF	
	F = 13.244 *p* < 0.001 R^2^ = 0.353 Adjusted R^2^ = 0.327		F = 19.037 *p* < 0.001 R^2^ = 0.440 Adjusted R^2^ = 0.417		F = 18.635 *p* < 0.001 R^2^ = 0.435 Adjusted R^2^ = 0.411		F = 10.516 *p* < 0.001 R^2^ = 0.303 Adjusted R^2^ = 0.274		F = 14.085 *p* < 0.001 R^2^ = 0.368 Adjusted R^2^ = 0.342		F = 48.717 *p* < 0.001 R^2^ = 0.668 Adjusted R^2^ = 0.654		F = 12.707 *p* < 0.001 R^2^ = 0.344 Adjusted R^2^ = 0.317		F = 19.955 *p* < 0.001 R^2^ = 0.452 Adjusted R^2^ = 0.429	
	ß	*p*	ß	*p*	ß	*p*	ß	*p*	ß	*p*	ß	*p*	ß	*p*	ß	*p*
	[95% CI]		[95% CI]		[95% CI]		[95% CI]		[95% CI]		[95% CI]		[95% CI]		[95% CI]	
PPIQ8	−0.191	0.015	−0.394	<0.001	−0.173	0.019	−0.175	0.032	−0.212	0.006	−0.390	<0.001	−0.040	0.610	−0.307	<0.001
	[−0.971, −0.138]		[−2.532, −1.212]		[−0.710, −0.208]		[−1.887, −0.340]		[−0.599, −0.169]		[−1.578, −0.903]		[−0.730, 0.106]		[−1.789, −0.765]	
PSEQ-HU	0.291	<0.001	0.225	<0.001	0.096	0.128	0.097	0.165	0.190	0.004	−0.180	<0.001	0.152	0.026	0.215	<0.001
	[0.243, 0.615]		[0.262, 0.851]		[0.050, 0.274]		[0.010, 0.700]		[0.081, 0.273]		[0.151, 0.452]		[0.112, 0.486]		[0.249, 0.705]	
Average NRS	−0.166	0.028	−0.130	0.063	0.052	0.460	−0.115	0.138	−0.079	0.287	−0.037	0.401	−0.186	0.014	−0.078	0.256
	[−2.937, −0.143]		[−4.292, 0.139]		[−0.444, 1.242]		[−4.316, 0.874]		[−1.042, 0.400]		[−4.664, −2.398]		[−2.950, −0.142]		[−2.573, 0.861]	
PSS14	−0.059	0.341	−0.030	0.599	−0.541	<0.001	−0.315	<0.001	−0.290	<0.001	−0.037	0.401	−0.372	<0.001	−0.229	<0.001
	[−3.242, 1.042]		[−4.892, 1.900]		[−4.423, −1.839]		[−14.585, −6.628]		[−2.619, −0.408]		[−2.650, 0.823]		[−5.985, −1.681]		[−6.712, −1.447]	
Age	−0.126	0.031	0.066	0.229	−0.002	0.968	−0.001	0.856	0.055	0.336	0.063	0.133	0.023	0.699	−0.034	0.523
	[−0.788, −0.028]		[−0.927, 0.278]		[−0.298, −0.160]		[−0.892, −0.519]		[−0.320, −0.072]		[−0.553, −0.063]		[−0.527, −0.236]		[−0.553, −0.381]	
BMI	−0.042	0.471	0.036	0.507	0.017	0.754	0.060	0.327	0.063	0.281	0.061	0.150	−0.101	0.088	0.098	0.072
	[−0.650, 0.293]		[−0.988, 0.508]		[−0.241, 0.328]		[0.493, 1.259]		[−0.113, 0.374]		[−0.108, 0.657]		[−0.865, 0.082]		[−0.065, 1.094]	
Educational level	0.085	0.144	0.010	0.846	−0.006	0.908	0.022	0.713	0.002	0.977	0.062	0.133	−0.050	0.386	0.059	0.267
	[1.697, 8.791]		[−7.886, 8.744]		[−4.829, 1.499]		[−11.402, 8.081]		[−3.381, 2.031]		[−1.273, 7.232],		[−9.231, 1.307]		[4.473, 8.417]	
Moderate physical activity	−0.027	0.647	0.033	0.539	0.015	0.776	−0.002	0.979	0.018	0.747	0.073	0.082	0.013	0.824	0.003	0.957
	[−0.026, 0.016]		[0.023, 0.044]		[−0.012, 0.014]		[−0.042, 0.036]		[−0.009, 0.012]		[−0.002, 0.032]		[−0.019, 0.023]		[−0.026, 0.026]	
Vigorous physical activity	0.057	0.313	0.033	0.529	−0.067	0.204	0.027	0.650	−0.010	0.859	0.034	0.397	0.085	0.135	0.111	0.034
	[−0.010, 0.031]		[−0.022, 0.043]		[−0.019, 0.006]		[−0.029, 0.047]		[−0.011, 0.010]		[−0.09, 0.024]		[−0.036, −0.005]		[0.02, 0.052]	

ß: Standardized regression coefficient (beta). (PF = Physical Functioning, RP = Role Limitations due to Physical Health, EW = Emotional Wellbeing, RE = Role limitations due to Emotional Problems, VT = Energy/Fatigue, BP = Bodily Pain, GH = General Health, SF = Social Functioning).

## Data Availability

The data presented in this study are available on reasonable request from the corresponding author. The data are not publicly available due to privacy restrictions.

## References

[1] Swift B., Taneri B., Becker C.M., Basarir H., Naci H., Missmer S.A., Zondervan K.T., Rahmioglu N. (2024). Prevalence, Diagnostic Delay and Economic Burden of Endometriosis and Its Impact on Quality of Life: Results from an Eastern Mediterranean Population. Eur. J. Public Health.

[2] Corte L.D., Filippo C.D., Gabrielli O., Reppuccia S., Rosa V.L.L., Ragusa R., Fichera M., Commodari E., Bifulco G., Giampaolino P. (2020). The Burden of Endometriosis on Women’s Lifespan: A Narrative Overview on Quality of Life and Psychosocial Wellbeing. Int. J. Environ. Res. Public Health.

[3] Parazzini F., Esposito G., Tozzi L., Noli S., Bianchi S. (2017). Epidemiology of Endometriosis and Its Comorbidities. Eur. J. Obstet. Gynecol. Reprod. Biol..

[4] Eisenberg V., Weil C., Chodick G., Shalev V. (2018). Epidemiology of Endometriosis: A Large Population-based Database Study from a Healthcare Provider with 2 Million Members. BJOG Int. J. Obstet. Gynaecol..

[5] Csákvári T., Pónusz-Kovács D., Kajos L.F., Elmer D., Pónusz R., Kovács B., Várnagy Á., Kovács K., Bódis J., Boncz I. (2023). Prevalence and Annual Health Insurance Cost of Endometriosis in Hungary—A Nationwide Study Based on Routinely Collected, Real-World Health Insurance Claims Data. Healthcare.

[6] Nnoaham K.E., Hummelshoj L., Webster P., d’Hooghe T., De Cicco Nardone F., De Cicco Nardone C., Jenkinson C., Kennedy S.H., Zondervan K.T. (2011). Impact of Endometriosis on Quality of Life and Work Productivity: A Multicenter Study across Ten Countries. Fertil. Steril..

[7] Soliman A.M., Rahal Y., Robert C., Defoy I., Nisbet P., Leyland N., Singh S. (2021). Impact of Endometriosis on Fatigue and Productivity Impairment in a Cross-Sectional Survey of Canadian Women. J. Obstet. Gynaecol. Can..

[8] Rossi V., Tripodi F., Simonelli C., Galizia R., Nimbi F.M. (2021). Endometriosis-Associated Pain: A Review of Quality of Life, Sexual Health and Couple Relationship. Minerva Obstet. Gynecol..

[9] Hunsche E., Gauthier M., Witherspoon B., Rakov V., Agarwal S.K. (2023). Endometriosis Symptoms and Their Impacts on the Daily Lives of US Women: Results from an Interview Study. Int. J. Womens Health.

[10] Vitale S.G., La Rosa V.L., Rapisarda A.M.C., Laganà A.S. (2017). Impact of Endometriosis on Quality of Life and Psychological Well-Being. J. Psychosom. Obstet. Gynecol..

[11] Mińko A., Turoń-Skrzypińska A., Rył A., Bargiel P., Hilicka Z., Michalczyk K., Łukowska P., Rotter I., Cymbaluk-Płoska A. (2021). Endometriosis—A Multifaceted Problem of a Modern Woman. Int. J. Environ. Res. Public Health.

[12] Kupec T., Kennes L.N., Senger R., Meyer-Wilmes P., Najjari L., Stickeler E., Wittenborn J. (2025). The Multifactorial Burden of Endometriosis: Predictors of Quality of Life. J. Clin. Med..

[13] Farenga E., Bulfon M., Dalla Zonca C., Tersar C., Ricci G., Di Lorenzo G., Clarici A. (2024). A Psychological Point of View on Endometriosis and Quality of Life: A Narrative Review. J. Pers. Med..

[14] Becker C.M., Bokor A., Heikinheimo O., Horne A., Jansen F., Kiesel L., King K., Kvaskoff M., Nap A., Petersen K. (2022). ESHRE Guideline: Endometriosis. Hum. Reprod. Open.

[15] Gambadauro P., Carli V., Hadlaczky G. (2019). Depressive Symptoms among Women with Endometriosis: A Systematic Review and Meta-Analysis. Am. J. Obstet. Gynecol..

[16] Škegro B., Bjedov S., Mikuš M., Mustač F., Lešin J., Matijević V., Ćorić M., Elveđi Gašparović V., Medić F., Sokol Karadjole V. (2021). Endometriosis, Pain and Mental Health. Psychiatr. Danub..

[17] Maulenkul T., Kuandyk A., Makhadiyeva D., Dautova A., Terzic M., Oshibayeva A., Moldaliyev I., Ayazbekov A., Maimakov T., Saruarov Y. (2024). Understanding the Impact of Endometriosis on Women’s Life: An Integrative Review of Systematic Reviews. BMC Womens Health.

[18] Dózsa-Juhász O., Makai A., Prémusz V., Ács P., Hock M. (2023). Investigation of Premenstrual Syndrome in Connection with Physical Activity, Perceived Stress Level, and Mental Status—A Cross-Sectional Study. Front. Public Health.

[19] O’Hara R., Rowe H., Fisher J. (2021). Self-Management Factors Associated with Quality of Life among Women with Endometriosis: A Cross-Sectional Australian Survey. Hum. Reprod..

[20] Kang H. (2021). Sample Size Determination and Power Analysis Using the G*Power Software. J. Educ. Eval. Health Prof..

[21] von Elm E., Altman D.G., Egger M., Pocock S.J., Gøtzsche P.C., Vandenbroucke J.P., STROBE Initiative (2008). The Strengthening the Reporting of Observational Studies in Epidemiology (STROBE) Statement: Guidelines for Reporting Observational Studies. J. Clin. Epidemiol..

[22] Eysenbach G. (2012). Correction: Improving the Quality of Web Surveys: The Checklist for Reporting Results of Internet E-Surveys (CHERRIES). J. Med. Internet Res..

[23] Chalmers K.J., Catley M.J., Evans S.F., Moseley G.L. (2017). Clinical Assessment of the Impact of Pelvic Pain on Women. Pain.

[24] Nicholas M.K. (2007). The Pain Self-Efficacy Questionnaire: Taking Pain into Account. Eur. J. Pain.

[25] Kovács-Szabó Z., Makai A., Ács P., Hock M. (2025). Validity and Reliability of the Hungarian Version of the Pain Self-Efficacy Questionnaire Among Women with Endometriosis and Chronic Pelvic Pain. Womens Health Rep..

[26] Cohen S., Kamarck T., Mermelstein R. (1983). A global measure of perceived stress. J. Health Soc. Behav..

[27] Stauder A., Konkoly Thege B. (2006). Az Észlelt Stressz Kérdőív (PSS) Magyar Verziójának Jellemzői. Mentálhig. Pszichoszomatika.

[28] Ware J.E., Sherbourne C.D. (1992). The MOS 36-Item Short-Form Health Survey (SF-36). I. Conceptual Framework and Item Selection. Med. Care.

[29] Czimbalmos Á., Zsolt N., Zoltán V., Husztik P. (1999). Páciens Megelégedettségi Vizsgálat SF-36 Kérdőívvel, a Magyarországi Normálértékek Meghatározása. Népegészségügy.

[30] Bató A., Brodszky V., Rencz F. (2025). Development of Updated Population Norms for the SF-36 for Hungary and Comparison with 1997–1998 Norms. Health Qual. Life Outcomes.

[31] Sima R.-M., Pleş L., Socea B., Sklavounos P., Negoi I., Stănescu A.-D., Iordache I.-I., Hamoud B., Radosa M., Juhasz-Boess I. (2021). Evaluation of the SF-36 Questionnaire for Assessment of the Quality of Life of Endometriosis Patients Undergoing Treatment: A Systematic Review and Meta-analysis. Exp. Ther. Med..

[32] Hayes M.H.S., Patterson D.G. (1921). Experimental Development of the Graphic Rating Method. Psychol. Bull..

[33] Jensen M.P., McFarland C.A. (1993). Increasing the Reliability and Validity of Pain Intensity Measurement in Chronic Pain Patients. Pain.

[34] Armstrong T., Bull F. (2006). Development of the World Health Organization Global Physical Activity Questionnaire (GPAQ). J. Public Health.

[35] Ács P., Betlehem J., Oláh A., Bergier B., Morvay-Sey K., Makai A., Prémusz V. (2020). Cross-Cultural Adaptation and Validation of the Global Physical Activity Questionnaire among Healthy Hungarian Adults. BMC Public Health.

[36] Rees M., Kiemle G., Slade P. (2022). Psychological Variables and Quality of Life in Women with Endometriosis. J. Psychosom. Obstet. Gynecol..

[37] Ang J.-Y., Leong E.-L., Chan H.-K., Shafie A.A., Lee S.-Q., Mutiah P., Lim R.V.-M., Loo C.-M., Rajah R.U.S., Meor Ahmad Shah M. (2022). Health-Related Quality of Life of Malaysian Patients with Chronic Non-Malignant Pain and Its Associated Factors: A Cross-Sectional Study. BMC Musculoskelet. Disord..

[38] Bandura A. (1977). Self-Efficacy: Toward a Unifying Theory of Behavioral Change. Psychol. Rev..

[39] Osaki J.D., Oliveira M.A.P. (2024). What Is Behind? Impact of Pelvic Pain on Perceived Stress and Inflammatory Markers in Women with Deep Endometriosis. J. Clin. Med..

[40] Facchin F., Barbara G., Saita E., Mosconi P., Roberto A., Fedele L., Vercellini P. (2015). Impact of Endometriosis on Quality of Life and Mental Health: Pelvic Pain Makes the Difference. J. Psychosom. Obstet. Gynecol..

[41] Márki G., Bokor A., Rigó J., Rigó A. (2017). Physical Pain and Emotion Regulation as the Main Predictive Factors of Health-Related Quality of Life in Women Living with Endometriosis. Hum. Reprod..

[42] Tennfjord M.K., Gabrielsen R., Tellum T. (2021). Effect of Physical Activity and Exercise on Endometriosis-Associated Symptoms: A Systematic Review. BMC Womens Health.

[43] Prémusz V., Makai A., Perjés B., Máté O., Hock M., Ács P., Koppán M., Bódis J., Várnagy Á., Lampek K. (2021). Multicausal Analysis on Psychosocial and Lifestyle Factors among Patients Undergoing Assisted Reproductive Therapy—With Special Regard to Self-Reported and Objective Measures of Pre-Treatment Habitual Physical Activity. BMC Public Health.

